# The debated neuroanatomy of the fourth ventricle

**DOI:** 10.1111/joa.13885

**Published:** 2023-05-12

**Authors:** Ronen Spierer

**Affiliations:** ^1^ Rappaport Faculty of Medicine Technion‐Israel Institute of Technology Haifa Israel

**Keywords:** fourth ventricle, inferior medullary velum, median aperture of Magendie, neuroanatomy, tela choroidea

## Abstract

The fourth ventricle is a small, fluid‐filled cavity located within the brain that plays a vital role in the body's physiological functions. Therefore, the anatomical elements forming it bear significant clinical relevance. However, the exact relations between the elements that form its roof are still debated in the neuroanatomical literature; the inferior medullary velum, and the ventricle's median aperture in particular. In some atlases, the inferior medullary velum is placed in the midline, while in others, it is placed in the transverse plane. The median aperture is also displayed in different ways in midsagittal drawings: as a round perforation of a midline velum, as a foramen in an uncharacterized part of the ventricle, and as a gap between the nodule and the brainstem. This work aims to provide a comprehensive review of the different descriptions of the fourth ventricle, in order to gain a clearer understanding of the ventricular system's structure.

## Graphical abstract

The fourth ventricle is a small, fluid‐filled cavity located within the brain that plays a vital role in the body's physiological functions and, therefore, the anatomical elements forming it bear significant clinical relevance. However, the exact relations between the elements that form its roof are still debated in the neuroanatomical literature; the inferior medullary velum, and the ventricle's median aperture in particular. This work aims to provide a comprehensive review of the different descriptions of the fourth ventricle, in order to gain a clearer understanding of the ventricular system's structure.

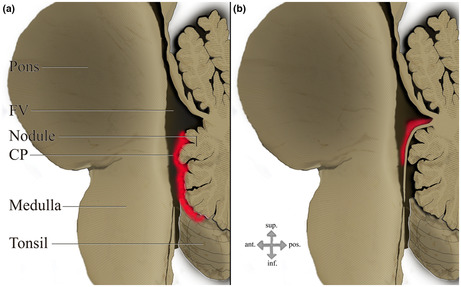

## 1 INTRODUCTION AND AIMS

The fourth ventricle is an intracerebral cavity that contains a small amount of cerebrospinal fluid (CSF), roughly 1 mL (Ertekin et al., 2012). It is tent‐like in shape: its base is formed, simply, by the rhomboid fossa of the brainstem. The roof of the ventricle is formed by multiple anatomical structures and is, therefore, more complex. CSF is drained to the ventricle through the cerebral aqueduct of Sylvius. In addition, the ventricle contains choroid plexus (CP) which produces some CSF (Strazielle & Ghersi‐Egea, 2000). Unlike the other three ventricles, the fourth ventricle plays a role not only in the production and drainage of CSF, but also in its ejection through three openings: the two lateral apertures (LA) of Luschka and the median aperture (MA) of Magendie (Johanson, 2008). These openings make the ventricle unique, both because it is not completely covered with ependyma and because it serves as a junction between the ventricular system and the subarachnoid space, where CSF is absorbed. This absorption is facilitated predominantly by the arachnoid villi, and secondarily by accessory pathways (Orešković et al., 2017). These villi are small protrusions of the arachnoid membrane that project into the venous sinuses. The arachnoid villi act as one‐way valves, allowing CSF to enter the venous sinuses, but not vice versa. This CSF absorption helps maintain the pressure inside the skull and is essential for the proper functioning of the central nervous system (Proulx, 2021).

CSF circulation, as well as the existence of the fourth ventricle, are not unique to humans (Wilder, 1893). However, the MA is absent in animals of many species, such as dogs, cats, rabbits, goats, opossums, and others (Coben, 1967). Cammermeyer described the MA in all apes he examined, and in rodents. Nevertheless, not in all species of rodents the aperture was present, and in the ones that had an open aperture, it structurally differed from the MA of primates. In all of these studied mammals, the LA were well developed (Cammermeyer, 1971).

In the embryo, the neural tube is formed during the fourth week of gestation. The rostral part of this tube later turns into five vesicles in which the four brain ventricles are formed. Three vesicles—the Mesencephalon, the Metencephalon, and the Myelencephalon develop into the brainstem, with the fourth ventricle and the outlet of the aqueduct as a remnant of the cavities inside them (Fotos et al., 2011; Shenoy & Lui, 2022). Simultaneously, the subarachnoid space is formed, but it is not yet connected to the ventricles. The connection between the fourth ventricle and the subarachnoid space is formed only a few days later (Sakka et al., 2011).

Blockage of the fourth ventricle is a medical emergency due to the potential for rapid accumulation of CSF and subsequent elevation in intracranial pressure, a condition termed hydrocephalus. This blockage usually happens when a neoplastic mass, a cyst, or hemorrhage occludes the ventricular circulation. Another important etiology of this condition is Arnold–Chiari malformation (Maller & Gray, 2016; Rekate, 2009; Shakeri et al., 2008). In addition, surgeries involving the fourth ventricle embed a risk of damaging vital brain functions due to its proximity to the brainstem. Accordingly, it is evident that the understanding of the anatomical structures of the fourth ventricle bears great medical and surgical importance.

### 1.1 The history of the fourth ventricle

The ventricular system has been known since the times of the ancient Greeks (Duque‐Parra et al., 2017). Hippocrates, who coined the term “hydrocephalus,” might have been aware of the existence of CSF (Aschoff et al., 1999). Later, Herophilus and Erasistratus, separately, were the first to describe the ventricular system (Engelhardt, 2018). Herophilus believed that since the ventricular system is the center of the brain, it is the seat of the mind. His greatest interest was in the fourth ventricle, which he believed was the body's “command center” (Gross, 1995; Stefanou, 2020).

A significant contribution for the study of the ventricular system was attributed by Galen. He proved by experiments that the four ventricles are connected, and was the first to describe the anatomy of the fourth ventricle in detail (Cucu et al., 2021). He also discovered an opening between the ventricular system and the subarachnoid space—the MA. However, this discovery was completely neglected (Torack, 1982). Galen likely understood the importance of the brain tissue, but still maintained that the soul resides in the ventricles, referring to the brain as merely a ventricular structure (Gross, 1995; Rocca, 1997).

Galen's findings in the Roman era were largely based on the dissection of animal cadavers, as the laws of the time prohibited the dissection of humans. Another significant progress of our understanding of the ventricular system was made by anatomists as Vesalius, who performed dissections on human cadavers, and later by Cotugno who gave the first reliable and adequate account of fluids found in human's ventricles (Pearce, 2004; Steele, 2014).

Haller, a contemporary of Cotugno, noticed that fluids could escape the ventricular system (Pearce, 2004). However, only during the nineteenth century, the French anatomist Magendie published his works on the ventricular system and rediscovered the opening between the fourth ventricle and the subarachnoid space (henceforth, the foramen of Magendie). Magendie wrote that CSF is moving back‐and‐forth through the MA. Magendie also made many other contributions such as giving CSF its name (Engelhardt, 2016; Magendie, 1842; Tubbs et al., 2008). Later, Bochdalek described the lateral recesses, which were later discovered to be leading to the LA by Luschka, who also confirmed the existence of the MA (Rogers & West, 1931). In the past, the term “metapore” for the MA was prevalent due to the aperture's relatively large size (Blake, 1900).

There was a debate regarding the truthfulness of Magendie's findings, however, this debate was not unique, as other components of the ventricular system were debated. For example, the terms “foramina of Monro” and “cerebral aqueduct of Sylvius” were described in the past as misnomers. Monro described the intraventricular foramina as connecting between the two lateral ventricles, whereas they actually constitute a connection between each lateral ventricle and the third ventricle (Sarwar, 1977). The cerebral aqueduct was attributed to Sylvius. Although there were two anatomists named Sylvius, and both published works about the aqueduct, neither were the first to describe it. Communication between the third and the fourth ventricle was known since the thirteenth century and possibly long before (Leite dos Santos et al., 2004; Stefanou, 2020). Thus, this communication was an established fact many centuries before communication between the fourth ventricle and the subarachnoid space was rediscovered by Magendie.

The question around the existence of the MA was a contentious topic and the center of a long academic dispute which continued in the twentieth century. Being described by Magendie as early as in 1828 (and by Galen 1600 years previous to him), some scientists did not agree with its existence; they believed that the ventricular system and the subarachnoid space were not connected at all (Rogers & West, 1931). These scientists, who denied the existence of the MA were Monroe, Krause, Todd, Virchow, Reichert, and others (Chapman, 1931; Reichert, 1861; Todd, 1847; Virchow, 1854). In a notable publication by Dandy from 1919, he wrote: “The existence of the foramen of Magendie is even now disputed by eminent authorities… Many still consider this foramen an artefact, as other foramina which have been described have proved to be.” (Dandy, 1919).

In 1875, Key and Retzius injected fluids into the ventricular system of cadavers, showing it passed through that opening and filled the subarachnoid space and vice versa, reinforcing its existence. The authors also concluded that CSF is absorbed in the subarachnoid space (Key & Retzius, 1875).

### 1.2 History of variations in drawings

Similar to the other three ventricles, the fourth ventricle has a long history that can be traced back to the ancient Greeks, with variations in its perception existing since that time. For example, Galen noted that some Greek anatomists misplaced the entire ventricle (Rocca, 2003).

As presented extensively later in this article, there were some variations in the placement of anatomical structures forming the fourth ventricle. Recently, Ciołkowski et al. (2011) described different variations of the MA in four anatomy atlases. Additionally, Johnston investigated whether the inferior medullary velum (IMV) was a single midline or a bilateral structure. Although most of the literature he reviewed described it as a midline structure, he found it to be horizontal and bilateral (Johnston, 1934). Roughly a century has passed since Johnston's publication, but most up‐to‐date atlases still insist on placing the IMV in the midline.

Regarding the fourth ventricle, the textbook of Clinical Neurology highlights its medical importance; however, it is illustrated variously in different drawings found in that book (Simon et al., 2015). Such illustrations are prevalent in anatomy textbooks and atlases, yet there is a significant inconsistency between them. Few inconsistencies are even found between different editions of the same book, and rarely, in the very same edition. In this article, we cover the literature for its variations in illustrating and describing the fourth ventricle.

## 2 ANATOMICAL ELEMENTS REVIEWED

There are two anatomical elements of the fourth ventricle that are varied in their description and illustration. The first one is the IMV, a neuron‐deprived structure of the flocculonodular lobe (Tubbs et al., 2013). To demonstrate the variations of this structure, a drawing by the renowned anatomy illustrator Netter might be a good example. It is a midsagittal brain section, published in 1953, displaying the IMV as a midline structure, supposedly a mirror image of the superior medullary velum, inserted in the fastigium (Netter, 1953). However, according to Johnston's histological findings, the IMV cannot be seen in such a dissection. Instead, the IMV is a paired transverse part of the flocculonodular lobe of the cerebellum that attaches the nodule to each flocculus (Johnston, 1934). Rhoton described the IMV as a butterfly‐shaped coronary membranous layer (Rhoton, 2000).

**FIGURE 1:**
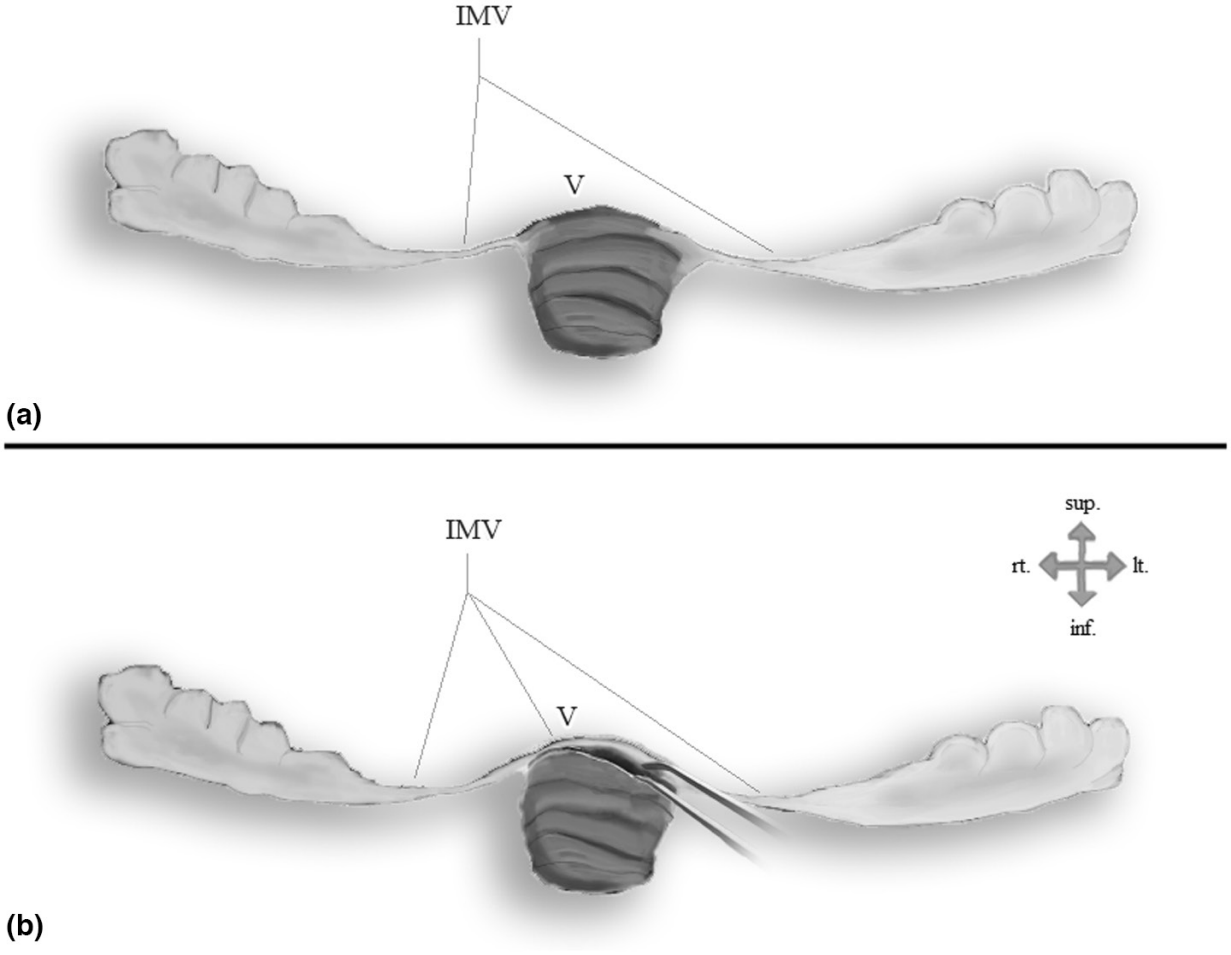
Anterior point‐of‐view of the flocculonodular lobe: (a) The inferior medullary velum is a bilateral horizontal structure, and the nodule is nakedly exposed to the fourth ventricle. (b) The inferior medullary velum is a midline structure, located anteriorly to the nodule, and separates it from the fourth ventricle. IMV, inferior medullary velum; V, ventricle.

More than that, Netter's drawing shows IMV as if it separates between the nodule and the fourth ventricle. However, other atlases presented the nodule as exposed to the ventricle and a forming part of its roof. The last of the two presentations is in agreement with Rhoton's definitions of the ventricle (Rhoton, 2000). Another drawing by Netter, which illustrates the anterior surface of the cerebellum, repeats this figuring of the IMV as partitioning between the nodule and the ventricle (Netter, 2018). In Sobotta's atlas, a drawing from the same point‐of‐view is present, and in contrast to Netter's drawing, the IMV is attached to each side of the nodule, but does not cover it (Paulsen & Waschke, 2018). These two variations in illustrating the flocculonodular lobe are shown in Figure 1.

Netter's original drawings, such as an axial section of the fourth ventricle, depicting the IMV as a midline structure, are included in Netter's Atlas of Neuroscience (Felten et al., 2022). This book also includes a new drawing by another physician‐illustrator, Craig. This axial drawing displays the vermis as standing out, nakedly, toward the ventricular space, thus contradicting Netter's drawings. Nevertheless, this part of the vermis is characterized as the uvula. However, according to Rhoton, this part is the nodule, as the uvula is placed in the cisternal wall (Rhoton, 2000).

The placement of the IMV is not the only element which varies between anatomy atlases. The foramen of Magendie is also drawn and described in different ways in the literature. According to Netter's drawings, this foramen is a pinhole in the IMV. Additionally, in Netter's illustration of a 3‐month‐old embryo's ventricular system, the MA is drawn as a planned round foramen in the roof plate (Felten et al., 2022). However, according to others, it is instead a gap between the nodule (or CP attached to the nodule), the tela choroidea (TC), and the rhomboid fossa of the brainstem (Blake, 1900; Ciołkowski et al., 2011). It can be said that this connection between the ventricle and the subarachnoid space is an aperture and not a foramen. Moreover, the dating of the formation of the MA also varies between textbooks. According to Netter's Atlas of Neuroscience, it is formed during the first trimester of pregnancy (Felten et al., 2022). According to Gray's Anatomy, it is formed during the second trimester (Fujii et al., 1980).

**FIGURE 2:**
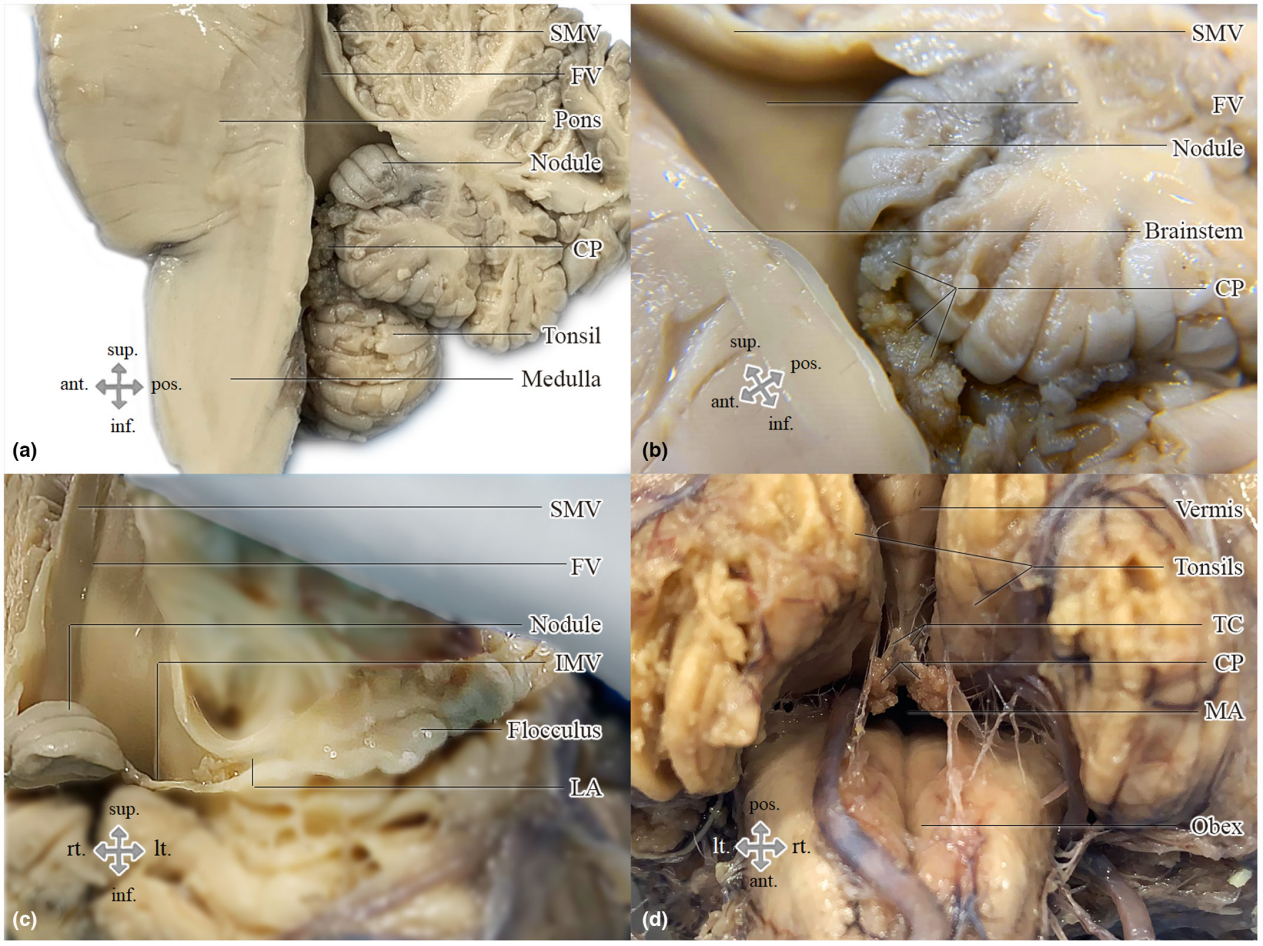
Photographs demonstrating the anatomical elements that form the fourth ventricle: (a) Midsagittal section of the hindbrain. (b) Closer view of the median aperture in the midsagittal section; the choroid plexus is attached to the vermis and passes through the median aperture. (c) Anterior view of the fourth ventricle, as the brainstem was retracted, which displays the flocculonodular lobe. The tela choroidea has been removed. The superior and inferior vela are perpendicular to each other. (d) Inferior view of a widely open median aperture, as the cerebellum and the brainstem were spread apart. The gap between the obex, the vermis, and the tela choroidea, in which the choroid plexus arises is the median aperture. CP, choroid plexus; FV, fourth ventricle; IMV, inferior medullary velum; LA, lateral aperture; MA, median aperture; SMV, superior medullary velum; TC, tela choroidea.

In Figure 2, pictures of the fourth ventricle are displayed. These are photographs of formalin‐fixed adult brains which were dissected in several ways in order to demonstrate the different anatomical elements of the fourth ventricle. The IMV is not seen in the median section (Figure 2a,b), but in the transverse plain, as the brainstem was pulled backward (Figure 2c). The MA, as seen in Figure 2d, is not a foramen, but rather a gap.

### 2.1 Early observations of the fourth ventricle

Since Magendie's research of the ventricular system in the 1820s, several works that characterized the fourth ventricle had been conducted. One such work was carried out by Johnston, which has been previously mentioned (Johnston, 1934). In Table 1, we provide a review of the methodology employed in these early works.

**TABLE 1:** Methodology of early works reviewed in this article.

Authors	Year	Number of human cadavers	Methods
Key and Retzius	1875	100	Gross anatomy and histology
Morton	1893	14	Gross anatomy
Blake	1900	N/A	Histology
Weed	1917	N/A	N/A
Chapman	1931	N/A	Histology
Rogers and West	1931	N/A	Gross anatomy
Johnston	1934	N/A	Gross anatomy and histology
Wilson	1937	1	Histology
Barr	1948	118	Gross anatomy
Hewitt	1960	38	Gross anatomy

While most of these studies described the MA in a similar way, some disputes were raised. For instance, Weed described the MA as a perforation in the pia mater of the TC. Wilson had a similar description. However, Morton described it as a broad slit between the cerebellum and the obex, emphasizing that it is not a small hole in the pia mater (Morton, 1893; Weed, 1917; Wilson, 1937).

Others described MA as a gap: According to Key and Retzius, the MA is a gap between the obex and the TC, as the latter has a thin prolongation that adheres to the uvula; the CP passes through the opening (Key & Retzius, 1875). Blake's observations were in agreement with this description (Blake, 1900). Barr, similarly to Hewitt, defined the borders as made by the obex and the pia‐ependymal roof, as the latter was attached to an undefined part of the inferior vermis (Barr, 1948; Hewitt, 1960).

Lastly, Rogers and West described the MA, not as a circular opening or aperture, but as a complete absence of the lower part of the roof extending downward from the IMV (Rogers & West, 1931).

## 3 REVIEW OF TEXTBOOKS AND ATLASES

Among the atlases reviewed in this study, the majority of them displayed the MA as a hole in the IMV, or as a hole in the uncharacterized inferior roof of the fourth ventricle. It is uncommon, yet in one atlas, a bewildering variation exists between a brain photograph and a drawing of the same slice presented on the same page. Especially, Snell's Clinical Neuroanatomy includes few midsagittal illustrations of the fourth ventricle: in ones, the MA is seen as an opening in the inferior roof. The CP is seen extended inferiorly through the roof. In others, it is depicted as a gap between the nodule and the brainstem, as the CP is continuous along the vermis and passes through the aperture into the cisterna magna (Splittgerber, 2019).

One of the reviewed drawings can be seen in both Schunke's atlas (Scheunke et al., 2016) and Gilroy's et al. atlas (Gilroy et al., 2020). These two atlases share the same publisher. The same drawing is also included in Sobotta's atlas (Paulsen & Waschke, 2018). Determining the correctness of this drawing, which is found a total of four times in these atlases, is beyond the scope of this article. However, it is important to mention that this drawing does not align with other illustrations.

This inconsistency in illustrating the fourth ventricle exists in anatomy textbooks as well. In a drawing that is included in Morris' Human Anatomy, the borders of the fourth ventricle are displayed in one fashion; the IMV is illustrated as a transverse bilateral element, and the MA appears to be a gap between the nodule and the brainstem in a midsagittal illustration. However, in another illustration found in this book, the MA is displayed as a perforation in the inferior roof. The last drawing is consistent with the written description (Jackson, 1923). Another example of a discrepancy between textual and graphical descriptions can be observed in Barr's Human Nervous System. While according to the text, the MA is a perforation in the IMV, an attached drawing does not illustrate the IMV, or other structure, as involving the MA (Kiernan & Rajakumar, 2014).

An interesting variation may be encountered when looking at Gray's Anatomy. In its current, 42nd edition, the roof of the fourth ventricle is described as follows: the IMV forms the inferior part of the roof, which separates the vermis from the ventricular cavity, and an opening in it creates the MA. A drawing, similar to Netter's, was added (Standring, 2021). Surprisingly, this description and associated illustration were not present in earlier editions of Gray's Anatomy. According to the 37th edition, the lower part of the roof is formed by the nodule, the IMV, the TC, the taenia and the obex; the IMV is described as “bilateral crescentic sheets flanking the nodule.” The drawings visualize the MA as a gap (Williams et al., 1989). In the 38th edition, while the drawings are the same, the MA is described as a perforation in the IMV (Williams, 1995). In the subsequent editions, figures were replaced with ones illustrating the MA as a hole in a midline IMV (Standring, 2005).

Another relevant description of the fourth ventricle is in the Waxman's textbook (Waxman, 2020). The IMV is described as forming the inferior part of the roof, and the MA is described as an opening in it. This description is almost identical, word for word, to the one found in an old textbook by Chusid, 1982. These two textbooks were published by the same publisher.

**FIGURE 3:**
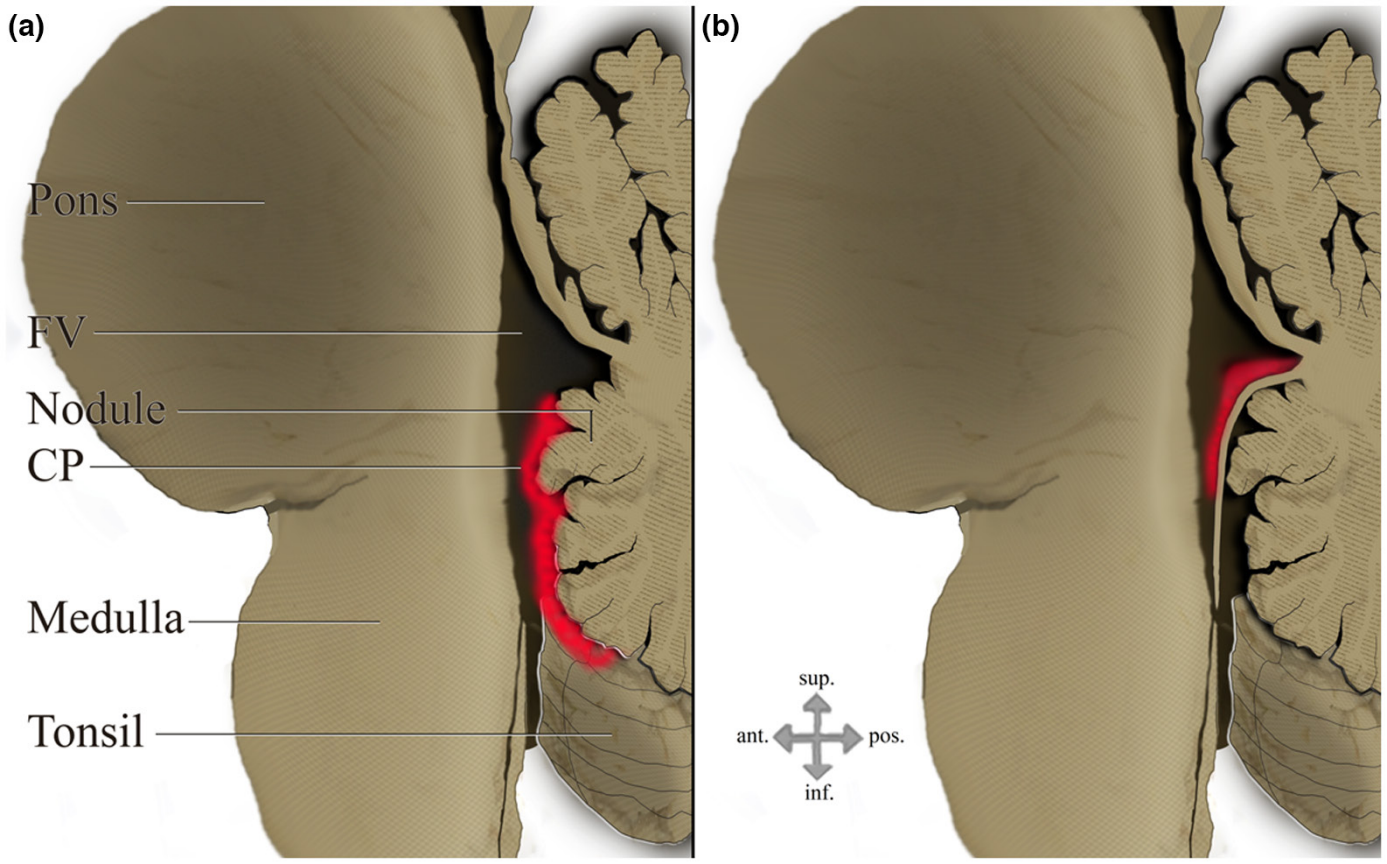
Midsagittal section of the fourth ventricle: (a) The inferior medullary velum is not seen, and the choroid plexus is continuous along the vermis through the median aperture. (b) The inferior medullary velum separates between the vermis and the fourth ventricle. A deficiency in the velum creates the median aperture. CP, choroid plexus; FV, fourth ventricle.

In Figure 3, the two variations in displaying a midsagittal section of the fourth ventricle are illustrated. Overall, we reviewed drawings from 24 atlases and textbooks. Each book is presented in Table 2, with a matching description. A total of 27 different drawings are described in the table: Six illustrated MA as a gap between the brainstem and the vermis and 21 illustrated it as a round opening or a perforation in the inferior wall of the ventricle. In 10 drawings, this wall was the IMV.

**TABLE 2:** Review of midsagittal drawings of the fourth ventricle.

Authors	Type of publication	Year	Description of the midsagittal illustration
Bourgery and Jacob	Atlas	1854	The median aperture is illustrated as in Figure 3(a)
Moeller and Reif	Atlas	2007	
Barr	Textbook	2014	
Chusid	Textbook	1982	The median aperture is illustrated as in Figure 3(b)
Heimar	Textbook	1983	
Tank and Gest	Atlas	2009	
Lindsay et al.	Textbook	2010	
Hendelman	Atlas	2016	
Rohen et al.	Atlas	2016	
Schunke et al.	Atlas	2016	
Dezena	Atlas	2017	
Netter	Atlas	2018	
Osborn et al.	Textbook	2018	
Simon et al.	Textbook	2018	
Sobotta	Atlas	2018	
Drake et al.	Atlas	2020	
Gilroy et al.	Atlas	2020	
Grant	Atlas	2020	
Waxman	Textbook	2020	
Martin	Atlas	2021	
Standring et al.	Textbook	2021	
Testut and Jacob	Textbook	1911	Both variations illustrated in Figure 2 are included in this atlas/textbook.
Morris	Textbook	1923	
Snell	Textbook	2019	

## 4 REVIEW OF CONTEMPORARY ARTICLES

Variations might still be evident among contemporary publications. For example, in a notable article published by Mussi and Rhoton, a presentation of the MA as a perforation in the TC takes place in a photograph of a cadaver (Mussi & Rhoton, 2000). This image contradicts other descriptions of the MA boundaries, such as the one provided by Ciołkowski et al., 2011. Nevertheless, it aligns with drawings from older publications of Rhoton (Fujii et al., 1980; Matsushima et al., 1982).

Mercier et al. described the MA as an opening in the IMV. Nevertheless, a split‐brain photograph that is included in that publication does not corroborate this description (Mercier et al., 2021). Similarly, illustrations presented in publications by Longatti et al., Chen and Haorah, and others are Netter‐styled as well (Cheng & Haorah, 2019; Longatti et al., 2008; Orešković & Klarica, 2010). In a review by Mortazavi et al., the MA is once again described as an opening in the IMV. They were probably based on a relatively new edition of Gray's Anatomy (Mortazavi et al., 2014). In a review of the ventricular system by Dezena et al., while the borders of the other three ventricles were described, the boundaries of the fourth ventricle lacked any reference (Dezena et al., 2020).

In contrary to this variation found in the literature, midsagittal drawings displaying the MA as a gap between the vermis and the brainstem, can also be found in few publications (Azab et al., 2014; Horsburgh et al., 2012).

## 5 DISCUSSION

An illustration of the brain from a midsagittal point‐of‐view is very common and is often seen in posters which may be found in health‐care facilities. Such illustrations are also found on the cover of textbooks. In these instances, variations in the fourth ventricle are not uncommon.

In their work, Ciołkowski et al. described the borders of the MA based on gross anatomy of cadavers. They also acknowledged that even though the borders were well defined by anatomists, various illustrations of the MA as a perforation in the roof of the fourth ventricle is still prevalent today (Ciołkowski et al., 2011). In that article, they reviewed the early works on the MA and deemed the differences between them insignificant. Contrarily, we did find these differences significant, as mentioned earlier in this article. For example, Rogers and West described MA as a complete absence of the lower part of the roof (Rogers & West, 1931). The other researchers mentioned by Ciołkowski et al. described MA as a gap between the obex, the TC, and the vermis, except for Rhoton, who described MA, simply, as an opening in the lower tip of the TC (Mussi & Rhoton, 2000; Rhoton, 2000).

Variations between anatomy atlases and even errors in the anatomical literature are not uncommon (Prestigiacomo, 2017). A publication by Corrales et al. reviewed inconsistencies in drawings of the cranial nerves (Corrales et al., 2017). Bogen described an error in another neuroanatomical structure—the thalamus. The error was, first, a significant reduction in the thalamic nuclei number, and second, misplacement of one of them. This mistake was titled “half a century perpetuating error” since there were about five decades between the first publication of the painting and the publication of Bogen's article (Bogen, 2006). Since then, even though the correct structure came to light, the illustration was not replaced or revised in the new editions of Netter's Anatomy. Overall, Bogen detailed several variations of the thalamus nuclei in anatomy books.

In contrary to the thalamic error, which according to Bogen was transmigrated from Netter's drawing into one famous textbook, and from it to others, we cannot point out any specific origin of the variations discussed in this article. However, we do know the variations described here preceded Netter, and that in some instances, one publication was based on another. For example, the aforementioned picture, showing the median aperture as an opening in the TC, published by Mussi and Rhoton (Mussi & Rhoton, 2000), was also used in another publication of Rhoton, and in two different textbooks (Chaichana & Quinones‐Hinojosa, 2019; Rhoton, 2000; Tubbs et al., 2022). Other pictures from that article were reprinted in articles by Salma et al., 2013 and Ghali, 2021. We also mentioned that in a review by Mortazavi et al., the authors relied on Gray's Anatomy in describing the inferior part of the roof as formed, simply, by the IMV. Gray's Anatomy in its turn is referring to another publication. However, in that publication, it was not written that the MA is an opening in the IMV (Mortazavi et al., 2014). Fujii et al. defined the borders of the inferior roof differently, as formed by the IMV, the TC, and the nodule, since they were relying on older edition of Gray's anatomy (Fujii et al., 1980). Mortazavi et al. also referred to a publication by Matsushima et al., which described this part of the roof as made by the vermis and the TC, in addition to the IMV (Matsushima et al., 1982).

In addition to these examples of imagery copying, we described a case of text being copied from one textbook to another, even though decades had passed between their publications. In this case, the description was different from known histological findings. We also mentioned that one drawing was published in three different atlases. Again, this drawing was observed to differ from anatomical findings, such as Chapman's.

It is possible that drawings illustrating the MA differently from its actual appearance originated from Magendie's description itself (Ciołkowski et al., 2011). However, it cannot be assumed with certainty that this was the source, if each variation ever had one. Perhaps, the presentation of the MA as a perforation in the inferior part of the roof was adopted by many anatomists sporadically due to its relative logical simplicity. With regard to the IMV, it can be hypothesized that few anatomists simply illustrated it as if it was a mirror image of the superior medullary velum. Another possible explanation is that a disambiguation between the IMV and the TC occurred. This explanation can be supported by an article by Wilson, in which he criticized the conclusions of Rogers and West. Among his disapprovals, he claimed that they failed to distinguish between the two elements, and consequently, described the borders of the MA incorrectly (Wilson, 1937). Wilson also found the need to specify that the term “inferior velum chorioidea,” used in the notable works of Weed, was referring to the lower part of the TC (Weed, 1917). This clarification teaches us the ease of mischaracterizing the IMV, which is not related to the MA.

The main objective of this article was not to answer the questions regarding the anatomy of the fourth ventricle, or to accurately define it; instead, our main purpose was to review the different ways in which it was described and illustrated. However, it is important to mention early histological studies that defined the anatomical elements that form the fourth ventricle: Johnston who found the IMV as a bilateral, not a midline, structure; Blake and Chapman who separately defined that the boundaries of the MA are the obex of the rhomboid fossa, the TC from each side, and the CP which is continuously attached to the vermis from its ventricular part to the cisternal. None of these works described the MA as bounded by the IMV (Blake, 1900; Chapman, 1931; Johnston, 1934). Therefore, and on the basis of our observations, we may conclude: The base of the fourth ventricle is formed by the rhomboid fossa; the upper part of its roof is formed by the superior medullary velum and the superior cerebellar peduncles; the lower part of the roof is formed by the nodule, the IMV, and the TC. After removing the choroid plexus, the MA is found to be bounded by the vermis, the TC, and the obex of the rhomboid fossa. The most accurate description and illustration of the fourth ventricle is to be found in the 37th edition of Gray's Anatomy (Williams et al., 1989), or in older editions.

It is acceptable that anatomists pay respect to tradition and history. One may find this habit positive; however, this practice may perpetuate long‐term errors, even years after they have been corrected. Furthermore, in other fields of science, avoiding the update of the study materials would be unsatisfactory. It is essential that students will be taught using reliable visual aids that match the anatomical features observed in cadavers.

## FUNDING INFORMATION

None.

## CONFLICT OF INTEREST STATEMENT

The authors report no potential conflicts of interest.

## Data Availability

Data sharing is not applicable to this article as no datasets were generated or analyzed during this study.
